# Anti-PD-1 and anti-PD-L1 drugs treatment-related adverse events for patients with cancer: Protocol for an overview of systematic reviews with meta-analyses

**DOI:** 10.1097/MD.0000000000016324

**Published:** 2019-07-05

**Authors:** Jing Li, Ming Liu, JianShu Wang, Zhao Liu, JinXu Xue, JianCheng Wang, JunHai Jia

**Affiliations:** a Gansu Provincial Cancer Hospital; b School of Basic Medical Sciences, Lanzhou University; c Gansu Provincial Hospital; d Hospital Management Research Center, Lanzhou University; e Gansu Provincial Hospital Rehabilitation Center, Lanzhou, China.

**Keywords:** adverse events, anti-PD-1/anti-PD-L1 drugs, cancer, evidence mapping, overview

## Abstract

**Background:**

Anti-programmed cell death 1 (PD-1) and anti-programmed cell death ligand 1 (PD-L1) drugs treatment-related adverse events (AEs) are not uniform based on current study for patients with cancer. The study aimed to provide a complete toxicity profile and toxicity spectrum for anti-PD-1 and anti-PD-L1 drugs.

**Methods:**

All systematic reviews (SRs) with meta-analyses (MAs) relate to the anti-PD-1 and anti-PD-L1 drugs and SRs will be searched in the database of PubMed, Embase, Cochrane Library, and Web of Science from inception to February 2019. Eligible publications must have reported site, organ, or system level data on treatment-related AEs. The following will extract from each SRs: first author, year of publication, country of origin, number of origin study, number of patients enrolled, participant characteristics, duration of cancer diagnosis, cancer types, detailed description of treatment, and occurrence of AEs. Preferred Reporting Items for Systematic Review and Meta-analysis (PRISMA) and A Measurements Tool to Assess Systematic Reviews 2 (AMSTAR-2) will be used to assess the reporting and methodological quality of SRs/MAs. The characteristics of the included SRs/MAs and their quality will descriptively summarized using systematically structured tables. A network meta-analysis (NMAs) approach versus a narrative synthesis will be used to examine data synthesis considered. Odds ratios and 95% credibility intervals will be used as summary statistics. Evidence mapping (EM) method will to present the evidence landscape related to anti-PD-1 and anti-PD-L1 drugs treatment-related AEs for patients with cancer.

**Discussion:**

The results of the overview will be submitted to a peer-reviewed journal for publication.

**Ethics and dissemination:**

Because this study is not a clinical study, and we will search and evaluate only existing sources of literature. So, ethical approval is not required.

## 1 Introduction

The immune system plays important roles in preventing cancer, such as suppresses virus-induced tumors, prompts resolution of cancer inflammation and eliminates tumor cells in certain tissues. Immune checkpoint inhibitors (ICI) can prompt the immune system to eliminate tumor cells.^[1]^ Over the past 10 years, ICI has been recognized as one of the most important breakthroughs in cancer therapy.^[2]^ They include 2 categories of agents which are Cytotoxic T-lymphocyte antigen 4 (CTLA-4) and Programmed cell death (PD-1) receptor and Programmed cell death ligands (PD-L1).^[3,4]^ Ipilumuab was the first checkpoint inhibitor approved for the treatment of advanced melanoma by the Food and Drug Administration in 2011.^[5]^ Now, other checkpoint inhibitors directed at the programmed death pathway are approved for the treatment of multiple cancers. Anti-programmed death pathway drugs include monoclonal antibodies directed at both PD-1 (nivolumab and pembrolizumab) and the PD-L1 (avelumab, atezolizumab, and durvalumab).^[6–10]^ These drugs all work by blocking the PD-1 or PD-L1 immune checkpoint pathway to reactivate T cell-mediated antitumor immunity.^[11]^ Therefore, although there provides impressive anti-tumor activity in many solid tumors, these have been reported to cause autoimmune-like disorders with reactivation of cellular immunity.^[12,13]^ Given the increasing use of anti-PD-1 and anti-PD-L1 drugs, understanding their toxicity profile and toxicity spectrum is crucial.

An overview of systematic reviews (SRs) that have been an increasingly popular method of evidence synthesis is an approach to synthesizing a large body of literature in a particular area.^[14,15]^ In recent years, there are a growing number of resources, including guidelines, recommendations, descriptions, and SRs, relating to overview methods.^[16–19]^ However, when the conclusion of SRs on an issue is inconsistent, the overview is still the best way to summarize the evidence.^[20]^

For all we know, some case report, clinical trial and SRs of PD-1 and PD-L1 drugs report treatment-related adverse events (AEs) and represent an ideal resource for comprehensive analysis of incidences of AEs. However, substantial variations exist in cancer type, drug and dosing schedule, and AEs reporting criteria in these studies. So, we performed an overview of treatment-related AEs/ meta-analyses (MAs) of the Food and Drug Administration–approved anti-PD-1 and anti-PD-L1 drugs in published SRs/MAs and to provide relevant personnel with more comprehensive and higher level evidence.

## 2 Methods

The content of this protocol follows the Preferred Reporting Items for Systematic Review and Meta-analysis Protocols (PRISMA-P) recommendations.^[21]^ This review has been registered on the International Prospective Register of Systematic Reviews (PROSPERO),^[22]^ with the registration number CRD42019126015. If protocol amendments occur, the dates, changes, and rationales will be tracked in PROSPERO.

### 2.1 Eligibility criteria

#### 2.1.1 Types of participants

People with cancer who are 18 years or older received anti-PD-1 and anti-PD-L1 drugs. Ignore the type of cancer and previous treatment therapy, such as surgery, radiation, and chemotherapy et al.

#### 2.1.2 Types of interventions

At least 1 of the study arms consisting of nivolumab or pembrolizumab or avelumab atezolizumab or durvalumab;

treatment-related AEs are caused by anti-PD-1 or anti-PD-L1 drugs or combine after compared all treatments.

#### 2.1.3 Types of outcome

We plan to select all probably treatment-related AEs and its grades as the primary outcome, because these were compatible with most SRs and deemed to be a suitable alternative to immune-related AEs. The Common Terminology Criteria for AEs is the most commonly used tool for evaluating AE type and severity in clinical practice with a grading scale and clear definitions. All grades, grade 3, and grade 4 AEs indicate complete, severe, and life-threatening toxicity, respectively.^[23]^

#### 2.1.4 Types of studies

SRs and MAs of data from randomized controlled trials (RCTs), nonrandomized controlled trials, observational studies, qualitative studies, case series, and case reports.

### 2.2 Search strategy

We will search the following electronic bibliographic databases from inception to February 2019: PubMed, Embase, Cochrane Library, and Web of Science. The search strategy will include only terms relating to describing anti-PD-1 or anti-PD-L1 drugs and SRs/MAs. The search terms will be adapted for use with other bibliographic databases in combination with database-specific filters, where these are available. The language will be restricted as English. The search strategy of PubMed as an example is shown in follow:

#1 nivolumab[Supplementary Concept] OR MDX-1106[Title/Abstract] OR ONO-4538[Title/Abstract] OR BMS-936558[Title/Abstract] OR Opdivo[Title/Abstract] OR nivolumab[Title/Abstract]

#2 pembrolizumab[Supplementary Concept] OR Pembrolizumab[Title/Abstract] OR MK-3475[Title/Abstract] OR Keytruda[Title/Abstract] OR Lambrolizumab[Title/Abstract] OR IBI308[Title/Abstract]

#3 avelumab[Supplementary Concept] OR Avelumab[Title/Abstract] OR bavencio[Title/Abstract] OR msb 0010682[Title/Abstract] OR msb 0010718c[Title/Abstract] OR msb 10682[Title/Abstract] OR msb 10718c[Title/Abstract]

#4 atezolizumab[Supplementary Concept] OR Atezolizumab[Title/Abstract] OR monoclonal antibody mpdl 3280a[Title/Abstract] OR rg 7446[Title/Abstract] OR tecentriq[Title/Abstract] OR tecntriq[Title/Abstract] OR MPDL3280A[Title/Abstract]

#5 Nivolizumab[Title/Abstract]

#6 durvalumab[Supplementary Concept] OR Durvalumab[Title/Abstract] OR MEDI4736[Title/Abstract] OR Imfinzi[Title/Abstract]

#7 B7-H1 Antigen[Mesh] OR B7-H1 Antigen[Title/Abstract] OR Programmed Cell Death 1 Ligand 1[Title/Abstract] OR B7-H1 Immune Costimulatory Protein[Title/Abstract] OR PD-L1 Costimulatory Protein[Title/Abstract] OR Programmed Cell Death 1 Ligand 1 Protein[Title/Abstract] OR CD274 Antigen[Title/Abstract] OR PDCD1 ligand 1[Title/Abstract] OR protein B7H1[Title/Abstract] OR protein PDCD1LG1[Title/Abstract] OR programmed death ligand 1[Title/Abstract] OR programmed deathligand 1[Title/Abstract] OR programmed death 1 ligand 1 antibody[Title/Abstract] OR programmed death 1 ligand 1 protein[Title/Abstract] OR programmed cell death Ligand-1[Title/Abstract] OR PDL1[Title/Abstract] OR PD 1[Title/Abstract] OR programmed cell death 1[Title/Abstract] OR programmed death 1 receptor[Title/Abstract] OR programmed death 1 receptor antibody[Title/Abstract]

#8 OR/1-7

#9 Network Meta-Analysis[Mesh] OR “Meta-Analysis as Topic“[Mesh] OR ”Meta-Analysis“[Publication Type]

#10 ”network meta analysis“[Title /Abstract] OR ”network meta analyses“[Title /Abstract] OR ”mixed treatment comparison meta analysis “[Title /Abstract] OR ”mixed treatment comparisons meta analyses“[Title /Abstract] OR ”mixed treatment meta analysis“[Title /Abstract] OR ”mixed treatment meta analyses“[Title /Abstract] OR ”mixed treatment comparisons“[Title/Abstract] OR ”mixed treatment comparison“[Title /Abstract] OR ”multiple treatment comparison meta analysis“[Title /Abstract] OR ”multiple treatment comparisons meta analyses“[Title /Abstract] OR ”multiple treatments meta analysis“[Title /Abstract] OR ”multiple treatments meta analyses“[Title /Abstract] OR ”multiple treatment meta analysis“[Title /Abstract] OR ”multiple treatment meta analyses “[Title /Abstract] OR ”multiple treatment comparison“[Title /Abstract] OR ”multiple treatment comparisons"[Title /Abstract]

#11 meta analysis[Title/Abstract] OR meta analyses[Title/Abstract] OR metaanalysis[Title/Abstract] OR metanalysis[Title/Abstract] OR met-analysis[Title/Abstract] OR metaanalyses[Title/Abstract] OR metanalyses[Title/Abstract] OR met-analyses[Title/Abstract] OR systematic review[Title/Abstract] OR systematic reviews[Title/Abstract]

#12 OR/9-11

#13 #8 AND #12

### 2.3 Study selection

**Figure 1:**
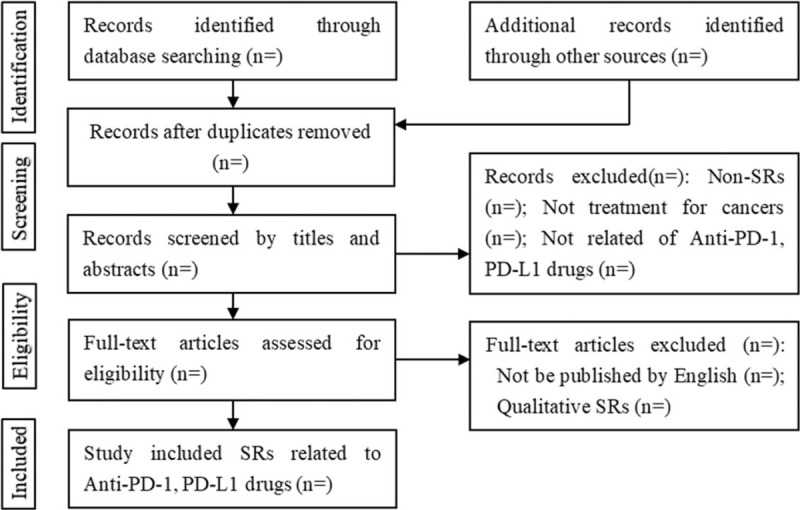
Selecting flowchart of systematic reviews related to anti-PD-1 and anti-PD-L1 drugs treatment-related adverse events. PD-1 = programmed cell death 1, PD-L1 = programmed cell death ligand 1.

Literature search records from electronic databases will be imported into the EndNote X8 literature management software (Thomson Reuters [Scientific] LLC, Philadelphia, PA). First, the titles and abstracts of all records will be reviewed independently by 2 reviewers. Then, full text of all potentially relevant literature will be retrieved for the inclusion or exclusion. All the works above will be done independently. Any conflict will be resolved by discussion. A flow diagram will be used to describe the process (Fig. 1).

### 2.4 Data extraction

Two reviewers will extract data independently using a predefined data extraction form. Disagreements will be resolved by discussion with a third reviewer. The following will extract from each embedded study: first author, year of publication, country of origin, number of origin study; the number of patients enrolled, participant characteristics, duration of cancer diagnosis, cancer types, detailed description of treatment, and occurrence of AEs. If the information could not be obtained from the published reports, then we will contact the review authors or authors of the original reports to provide clarification and further details.

### 2.5 Quality assessment

We plan to address 2 different quality assessments in this overview: the reporting quality and the methodological quality of the SRs. Two review authors to assess quality independently. Discrepancies can be resolved through discussion or, if required, consulate by the third person.

#### 2.5.1 Reporting quality assessment

The Preferred Reporting Items for Systematic Review and Meta-analysis (PRISMA) checklist including 7 parts with 27 items.^[24]^ The developers of the PRISMA proposed 3 answer options for each item: yes, no and partial. We will use PRISMA checklist to assess each item of the included SRs. Each of the items will be scored “1” for yes, “0.5” for partial, and “0” for no. We will judge the reporting quality based on the score of each SRs.

#### 2.5.2 Methodological quality assessment

The methodological quality of the included SRs/MAs will be assessed by the ‘A Measurement Tool to Assess Systematic Reviews’ (AMSTAR) 2 instrument. This updated version of the original AMSTAR tool allows for the appraisal of SRs of randomized and non-randomized studies of interventions.^[25]^ We will evaluate each review against the 16-item instrument. An overall rating of quality will be assigned according to the algorithm suggested by Shea et al^[25]^ A Measurements Tool to Assess Systematic Reviews 2 (AMSTAR-2) classifies the quality of an SR as high, moderate, low, or critically low, based on 16 domains, 7 of which are critical (items: 2, 4, 7, 9, 11, 13, and 15) and 9 of which are non-critical. A review without a critical flaw is classified as being of high or moderate quality according to the number of non-critical weaknesses; a review with no or 1 non-critical weakness will be classified as being of high quality, and others, as being of moderate quality. A review with 1 critical flaw will be classified as being of low quality and 1 with more than 1 critical flaw is classified as being of critically low quality. The overall quality of each outcome will be judged according to the summary of the quality of the included reviews.

### 2.6 Data synthesis

The characteristics of the included studies and their quality will be descriptively summarized using systematically structured tables. We will examine data synthesis considered in terms of a network meta-analysis (NMA) approach versus a narrative synthesis. We will consider the implementation of indirect comparisons and NMA in this overview from 2 different perspectives:

(1) overview and detail, based on the total number of all treatment-related AEs and the number of each specific treatment-related AE, respectively.
(2) Regardless of the AEs grading, general safety will be used to indicate the overview of treatment-related AEs with distinguishing between their specific classifications.

We will use odds ratios and 95% credibility intervals as summary statistics to quantify the effect of dose (of anti PD-1 and anti-PD-L1 drugs) or drug on the risk of grade 1–5 and grade 3 or 4 AEs in the NMA. Odds ratios greater than 1 represented a safety benefit favoring the control group. Two-sided *P* <.05 was considered significant. If an SR reported 0 AEs in any treatment, the classic half-integer continuity correction (adding a 0.5 to each cell) will be applied for data preparation. We will apply R (version 3.4.1; R Foundation for Statistical Computing, Vienna, Austria) software to perform the calculations. If network analysis should not possible, we will not conduct a quantitative analysis but undertake a qualitative synthesis. As far as possible, we will rely on data reported in the individual SRs. In rare cases, we anticipate that it may be necessary to reanalyze the data so comparable data are presented in the overview.

### 2.7 Subgroup analysis

If the necessary data are available, subgroup analyses will be done for specific cancer types (lung cancer, melanoma, etc).

### 2.8 Evidence mapping

We will use Evidence mapping (EM) method to present the evidence landscape related to anti-PD-1and anti-PD-L1 drugs treatment-related AEs for patients with cancer.^[26,27]^ The presentation form of EM will use the bubble plot and, which will display information on 3 dimensions: the AEs grading (y-axis), such as all grade AEs, grade1–2 AE, grade3–4 AE, and Death; the type of AEs (Colitis, Hepatitis, Pneumonitis, Hypothyroidism, and Rash etc) of the anti-PD-1 and anti-PD-L1 drugs treatment-related (x-axis); the number of primary studies included in each SRs/MAs (each bubble and bubble size). In addition, each bubble is also a pie chart that shows the proportion of RCTs included in the SRs/MAs through a black bold line.

## 3 Discussion

Immunotherapy, as a drug class, boosts the body's natural defense against cancer. Anti-PD-1 and anti-PD-L1 drugs are overall less toxic than standard chemotherapy, but a lot of clinical and SRs have found some immune-related AEs, such as colitis, hepatitis, pneumonitis, and hypothyroidism as well as more general AEs related to immune activation, including fatigue, diarrhea, and rash, have been common.^[8,28]^ However, the conclusions of these studies are not uniform. So, we plan to report a comprehensive dose and drug-based overview of the comparative safety of anti-PD-1and PD-L1 drugs, with the aim of providing a complete toxicity profile and toxicity spectrum of anti-PD-1/anti-PD-L1 drugs alone or in combination with conventional therapy.

## Author contributions

Jing Li, Ming Liu, and JunHai Jia conceived the study. JinXu Xue and JianCheng Wang designed the search strategy and flowchart of literature selection. JianShu Wang and Zhao Liu participated critical revision of the manuscript. All the authors contributed to draft and approve the study protocol. JunHai Jia will supervise the overall conduct of the study. Conceptualization: Jing Li, Ming Liu, JunHai Jia. Investigation: Ming Liu. Methodology: JianShu Wang, Ming Liu Resources: Zhao Liu, JinXu Xue, JianCheng Wang Writing-original draft: Jing Li. Writing-review and editing: JunHai Jia, JianShu Wang, Zhao Liu.

**Conceptualization:** Ming Liu, Jing Li, JunHai Jia.

**Investigation:** Ming Liu.

**Methodology:** Ming Liu, JianShu Wang.

**Resources:** Zhao Liu, JinXu Xue, JianCheng Wang.

**Writing – original draft:** Jing Li.

**Writing – review & editing:** JianShu Wang, Zhao Liu, JunHai Jia.
